# Potential Functions of the BMP Family in Bone, Obesity, and Glucose Metabolism

**DOI:** 10.1155/2021/6707464

**Published:** 2021-06-23

**Authors:** Yao Chen, Bingwei Ma, Xingchun Wang, Xiaojuan Zha, Chunjun Sheng, Peng Yang, Shen Qu

**Affiliations:** ^1^Chengdu Second People's Hospital, Chengdu 610017, China; ^2^Department of Gastrointestinal Surgery, Shanghai Tenth People's Hospital Affiliated to Tongji University, Shanghai, China; ^3^Thyroid Research Center of Shanghai, Shanghai 200072, China

## Abstract

Characteristic bone metabolism was observed in obesity and diabetes with controversial conclusions. Type 2 diabetes (T2DM) and obesity may manifest increased bone mineral density. Also, obesity is more easily to occur in T2DM. Therefore, we infer that some factors may be linked to bone and obesity as well as glucose metabolism, which regulate all of them. Bone morphogenetic proteins (BMPs), belonging to the transforming growth factor- (TGF-) beta superfamily, regulate a diverse array of cellular functions during development and in the adult. More and more studies revealed that there exists a relationship between bone metabolism and obesity as well as glucose metabolism. BMP2, BMP4, BMP6, BMP7, and BMP9 have been shown to affect the pathophysiological process of obesity and glucose metabolism beyond bone metabolism. They may exert functions in adipogenesis and differentiation as well as insulin resistance. In the review, we summarize the literature on these BMPs and their association with metabolic diseases including obesity and diabetes.

## 1. Introduction

The number of obesity and related metabolic disorders including type 2 diabetes (T2DM) is increasing worldwide with the changes in lifestyles [1]. Osteoporosis is also common, especially in women over 55 years old and men over 65 years old [2]. Osteoporosis is characterized by low BMD and microarchitectural deterioration of bone tissue leading to decreased bone strength and increased risk of fractures. The dual-energy X-ray absorptiometry (DXA) method is standard for determining BMD. However, DXA is a two-dimensional technique which cannot be used to measure true volumetric BMD and bone size or to separate trabecular from cortical bone. DXA cannot be used to measure bone microstructure or quality, which influences fracture risk [3]. Therefore, it is not capable of accurately assessing the fracture risk [4].

Bone mineral density (BMD) in obesity and T2DM is controversial, and the interaction between obesity, glucose metabolism, and bone metabolism is complexly linked to many mechanical and biochemical factors [5]. More and more studies revealed that there exists a relationship between bone metabolism and obesity as well as glucose metabolism. Bone morphogenetic proteins (BMPs), a member of the transforming growth factor- (TGF-) *β* superfamily, which can be divided into 20 kinds of BMP are widely recognized. It has multifunctions that far exceed those of other members of the TGF family. BMP as a secreted protein is linked to many pathologies, including obesity and diabetes as well as related complications. The BMP family plays a role as a bridge between glucose metabolism and bone metabolism.

The central role of BMPs in the remodeling process of the human skeleton has been identified in numerous experimental and clinical studies [6]. BMPs have been shown to promote the osteogenic differentiation of murine multilineage cells (MMCs) and to promote bone formation in bone diseases [7]. BMPs appear to be key agents in the osteoblastic differentiation of mesenchymal stem cells, and more recent evidence implicates them to exert functions in the cells of the osteoclastic lineage [6]. In vitro studies demonstrate that exogenous BMP activation increases osteoclastogenesis. BMP2, BMP4, BMP6, and BMP7 have been studied in the context of osteoporosis and have been associated with its pathophysiological pathways [6]. They complete these actions through both SMAD-dependent and SMAD-independent signaling pathways. BMP ligands interact with combinations of type 1 and type 2 receptors, which, in turn, activate effectors called receptor-activated- (RA-) SMADs. They translocate to the nucleus and accomplish gene regulation of genomic DNA.

In addition, BMPs are important regulators of adipogenesis and may play a role in obesity. Obesity is accompanied by an increase in both adipocyte number and size. The increase in adipocyte number is the result of recruitment to the adipocyte lineage of pluripotent stem cells present in the vascular stroma of adipose tissue. BMPs' signaling pathway has a dominant role in adipocyte lineage determination [8]. Also, they are related to obesity-related metabolic disorders including glucose metabolism [9] [10]. A research showed that BMP2 is correlated with diabetic status [10]. BMPs increase glucose uptake in mature 3T3-L1 adipocytes by PPAR*γ* and GLUT4 upregulation [11].

**Figure 1:**
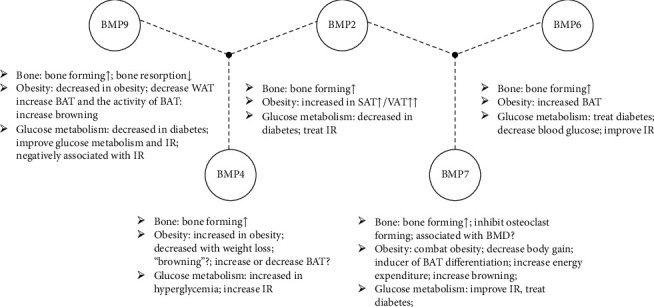
Effects of BMPs (BMP9, BMP4, BMP2, BMP7, and BMP6) on bone, obesity, and glucose metabolism. BMP: bone morphogenetic protein; WAT: white adipose tissue; BAT: brown adipose tissue; IR: insulin resistance; SAT: subcutaneous adipose tissue; VAT: visceral adipose tissue; BMD: bone mineral density.

In this review, we summarize the BMPs which both affect bone and obesity as well as glucose metabolism to better understand the potential link between bone metabolism, obesity, and type 2 diabetes. These effects of BMPs (BMP9, BMP4, BMP2, BMP7, and BMP6) on bone, obesity, and glucose metabolism are shown in Figure 1.

## 2. Bone Metabolism in Obesity and Type 2 Diabetes

### 2.1. Bone Metabolism and Obesity

The interaction between obesity and bone metabolism is complexly related to many mechanical and biochemical factors [5]. The traditional view pointed out that obesity is protective against fractures. BMD in patients with obesity was significantly higher than that in the lean Chinese population [12]. However, another study finds that sarcopenic obesity is associated with the development of osteoporosis among the middle-aged and elderly Korean population [13]. Another study shows a negative relationship between waist circumference which represented abdominal obesity and BMD in the femoral neck and total hip [14]. In addition, the positive effects of body weight on BMD cannot counteract the detrimental effects of obesity on bone quality [15].

Besides, interventions of reducing body weight likely lead to bone loss over time. For example, the effects of bariatric surgery on BMD are dependent on the type of surgical procedure [5]. A meta-analysis regarding bone loss after bariatric surgery showed that BMD at the femoral neck decreased after bariatric surgery, compared to that in nonsurgical controls, while BMD at the lumbar spine did not show a difference between groups [16]. Change in bone occurring in obese men has been evaluated, with the results that found that femoral neck and total hip BMD decreased and spine BMD increased significantly after laparoscopic sleeve gastrectomy (LSG) [17].

### 2.2. Bone Metabolism and Diabetes

BMD in patients with T2DM is controversial. Individuals with diabetes have a higher or normal BMD compared with those without diabetes while with a higher fracture risk [18, 19]. The study finds that the risk of fragility fractures is increased in patients with either type 1 diabetes mellitus or T2DM [20]. The underlying mechanism may involve bone turnover which is decreased, and the bone material properties and microstructure of bone are altered in both diabetes [20]. Insulin deficiency in T1DM or loss of incretin effect in T2DM impaired the bone health as hyperglycemia impairs osteoblast function, generates abnormal modifications of the bone protein matrix, induces a state of chronic inflammation, and increases the risk of falls and fractures [21]. Also, associated factors create a milieu that promotes MSC fate toward adipogenesis over osteoblastogenesis which leads to a low bone turnover phenotype [21]. A large registry-based study showed that diabetes is associated with slightly greater BMD loss at the femoral neck but not at other parts of measurements including the lumbar spine or total hip when adjusted for both age and BMI [22].

Diabetic patients are at increased risk of fragility fractures, while the mechanism of bone fragility in these patients is likely multifactorial [23]. Data from the Osteoporotic Fractures in Men (MrOS) study were analyzed, with the results that showed that men with diabetes who are using insulin have an increased risk of nonvertebral fracture [24]. Another study showed that T2DM was not associated with higher prevalent or incident vertebral fractures in older men after adjustment for BMI and BMD [25]. Patients with T2DM have a higher fracture risk compared with nondiabetics, despite having higher BMD. However, greater insulin resistance is not found to be associated with increased fracture risk after adjustment for BMI and BMD, which is in contrast to the relationship between T2D and fracture risk [26].

BMD is measured by dual X-ray absorptiometry (DXA). However, BMD evaluation by DXA may be inadequate for evaluating the risk of fracture in the endocrine-related forms of osteoporosis such as obesity and diabetes [19]. Some new tools of noninvasively estimating bone quality have been done in the clinical practice for optimizing the fracture risk [27]. Overall, bone metabolism in obesity and diabetes needs to be further studied, and the factors affecting all of them need to be investigated and clarified.

## 3. BMPs and Bone

### 3.1. BMP9

BMP9 (also known as growth and differentiation factor- (GDF-) 2), as a member of the BMP family, which is secreted by nonhepatic parenchymal cells has a similarity of 50~55% with other BMPs including BMP2, BMP4, BMP5, BMP6, BMP7, and BMP8 [28]. And it plays multiple functions in humans including iron metabolism, chondrogenesis, neuronal differentiation, angiogenesis, glucose, and lipid metabolism except for inducing osteogenesis and chondrogenesis [9, 29–31].

Bone remodeling is tightly regulated through both bone resorption dominated by osteoclasts and bone formation dominated by osteoblasts with a dynamic process. Imbalance of bone remodeling may lead to pathological conditions, such as osteoporosis. BMP9 predominantly produced in the liver has dual regulatory effects on bone remodeling. A study implemented in an ovariectomy mouse model found that BMP9 attenuates bone loss and improves bone biomechanical properties in vivo by increasing bone-forming activity and suppressing bone resorption activity [32]. BMP9 is an important factor in bone formation [7, 33, 34]. BMP9 significantly mediated callus formation and increased bone mass and strength in osteoporotic rats [33]. It increases the expression of mRNA levels of the osteoblast differentiation markers, such as ALP, Cola1, and OCN in MC3T3-E1 cells by upregulating LGR6 and activating the Wnt/*β*-catenin pathway [32]. The expression of two key transcription factors (OSX and RUNX2) that regulate the target genes of osteoblastic differentiation was increased by BMP9 intervention [32].

Meanwhile, BMP9 suppresses receptor activator of nuclear factor-*κ*B (NF-*κ*B) ligand- (RANKL-) induced osteoclast differentiation of bone marrow macrophages (BMMs) by inhibiting the Akt-NF-*κ*B-NFATc1 pathway [32]. Therefore, BMP9 may be explored as an effective therapeutic strategy for osteoporosis [35–38].

### 3.2. BMP4

Bone loss in osteoporosis is caused by an imbalance between resorption and formation on endosteal surfaces of trabecular and cortical bone. BMP4 is identified as a bone-inducing factor with an important role in bone formation [39]. Injection of BMP4-transduced MSCs in mice induced bone formation [40]. Trabecular BMD determined by pQCT increased 20.5% at 14 days, and total BMD increased 6.5% at 14 days and 10.4% at 56 days in these animals [40]. BMP4 stimulates the synthesis of osteocalcin and osteoprotegerin via activation of the P38 MAPK signaling pathway in osteoblasts [41].

The BMP4 gene is associated with hip BMD in postmenopausal women, which is presumably via the regulation of anabolic effects on the skeleton [42]. The ex vivo gene therapy could be a promising tool for the treatment of osteoporotic fractures. Primary muscle-derived cells were isolated from the hindlimb muscle of rats and retrovirally transduced to express BMP4, with the results that found that the bone healing process in the osteoporotic bone was improved to the level similar to that of normal bone [43].

### 3.3. BMP2

BMP2 is used to augment bone formation, which is similar to other BMPs that are well known as osteogenic growth factors [44]. Recombinant human BMP2 significantly stimulated bone formation in diabetic animal and enhanced bone regeneration in normal animals which means that BMP2 may be beneficial in treating the deficient intramembranous bone formation in diabetes [45].

Sclerostin, an osteocyte product, is encoded by the SOST (sclerosteosis) gene on chromosome 17 in humans, inhibits bone formation, and therefore is an important regulator of bone mass [46, 47]. It binds to the LRP5/6 receptor and frizzled coreceptor on the osteoblast cell surface, thereby interfering with Wnt ligand binding and hence blocking osteoblast differentiation and activity [48]. It decreases osteoblast activity while maintaining osteoclast function which leads to a shift of the bone remodeling balance towards bone resorption and bone loss. Therefore, targeting sclerostin expression could be a valuable tool for the prevention of osteoporosis.

BMPs increase sclerostin levels [47]. Administered BMPs induce bone formation at osteoblastic levels and with the parallel induction of sclerostin expression preventing overstimulation of the anabolic processes or ectopic bone formation. The study found that high BMP2 doses stimulate sclerostin expression in a negative feedback loop to prevent bone overgrowth and ectopic bone formation through the Wnt signaling pathway [48].

### 3.4. BMP7

BMP7 also known as osteogenic protein-1 (OP-1) is well established as having the osteoinductive activity. BMP7 mediated osteoblastic differentiation by recruiting stem cells to injured sites and induces osteoblast proliferation [49]. In addition, BMP7 inhibits osteoclast formation from monocyte precursor cells in vitro by interfering with signaling pathways [50]. Association of BMP7 and BMD was also investigated. BMP7 gene polymorphisms are associated with BMD in 920 European Americans. However, the common genetic polymorphisms of the BMP7 gene are not major contributors to variations in BMD or osteoporotic fracture in postmenopausal Chinese women.

Overall, better understanding of the mechanism of the BMP family on bone formation may help to prevent the development of osteoporosis.

## 4. BMPs and Obesity

### 4.1. BMP9

#### 4.1.1. BMP9 in Humans with Obesity

In humans, a previous study has shown that plasma BMP9 concentrations were significantly associated with metabolic syndrome (Mets) even after controlling for anthropometric variables and lipid profiles, and its levels were significantly lower in 362 newly diagnosed patients with Mets compared to the healthy controls [9]. Obesity is an important component of the Mets. Plasma BMP9 levels were associated with the key components of Mets such as obesity, and its levels reduced progressively with an increasing number of Mets components [9]. And the best cutoff value for circulating BMP9 levels to predict Mets was 56.6 ng/L in humans [9]. Besides, plasma BMP9 was associated negatively with the waist-hip ratio (WHR) which represents abdominal obesity in these patients with Mets [9].

#### 4.1.2. BMP9 and Adipose Tissue

In an animal experiment, the administration of BMP9 into obese mice expresses enhanced gene expression of fibroblast growth factor 21 (FGF21), which is a metabolic regulator, and reduced a spectrum of pathological symptoms caused by high-fat diet- (HFD-) induced obesity [51]. Besides, BMP9 is effective to treat obesity-mediated nonalcoholic fatty liver disease (NAFLD) [51]. The animal study showed that BMP9 alleviated hepatic steatosis, serum levels of alanine aminotransferase (ALT), and total cholesterol [51]. BMP9-enhanced expression of FGF21 reduces a serum level of ALT as well as cholesterol. Fibroblast growth factor 21 (FGF21) is predominantly released from hepatocytes. FGF21 can induce fatty acid oxidation in WAT and suppress lipogenic genes in the liver to reduce triglyceride accumulation.

Brown adipose tissue (BAT), characterized by the expression of the thermogenic uncoupling protein 1 (UCP1), has recently been described in adult humans [52]. The effect of BAT on thermogenic activity is mainly controlled by the sympathetic nervous system [52]. Therefore, stimulation of BAT activity and/or recruitment of UCP1-positive cells are relevant targets for the treatment of obesity in humans [52]. A study has proven that BMP9 has a role in brown adipogenesis and suppressing pathophysiology of HFD-induced obesity in vitro [53]. BMP9 induced gene expression of uncoupling protein 1 (UCP1) and cell death-inducing DNA fragmentation factor-like effector A (CIDEA) but not in the visceral adipose tissues from the mice fed with HFD [53]. BMP9 leads to the activation of BAT thermogenesis, as well as to “browning” of white adipose tissue (WAT) [52].

Besides, systemic intraperitoneal injection of a recombinant BMP9 derivative suppressed weight gaining of high-fat diet-induced obese mice by reducing the sizes of white adipocytes [53]. The underlying mechanism of this effect may involve the activing receptor-like kinase 1 signaling pathway and enhance fatty acid synthase expression in the liver of obese mice [53].

**Figure 2:**
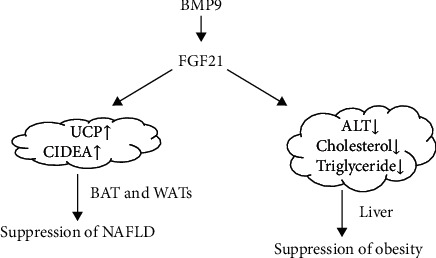
BMP9 enhanced expression of FGF21 reduces a serum level of ALT as well as cholesterol and enhances brown adipogenesis, resulting in suppression of NAFLD and obesity.

Overall, BMP9 is closely associated with obesity, mainly reducing the bodyweight of decreasing WAT, enhancing the activity of BAT, and increasing BAT as well as “browning.” It may be a method for the treatment of obesity and obesity-related complications such as NAFLD as presented in Figure 2.

### 4.2. BMP4

#### 4.2.1. BMP4 Levels in Patients with Obesity

A previous study also showed a relationship between BMP4 and obesity [54]. Serum BMP4 levels were significantly increased in subjects with obesity or Mets [55]. Kim et al. showed that serum BMP4 levels were decreased at 1 year after RYGB in 57 obese patients with diabetes [56]. Besides, serum BMP4 was proven to decrease at 3 and 6 months after LSG in females with obesity along with decreased BMD [57].

#### 4.2.2. BMP4 and Adipose Tissue

BMP4 is secreted by adipose cells and increases in hypertrophic obesity which plays a key role in regulating adipogenic precursor cell commitment and differentiation [58]. BMP4, secreted by WAT, is an integral feedback regulator of both white and beige adipogenic commitment and differentiation [58]. Increased BMP4 in adipose cells preferentially regulates the beige/brown phenotype [58]. BMP4 plays a role in brown adipocyte formation and activity. It has a dual function in adipogenesis by inducing adipocyte commitment while inhibiting the acquisition of a brown phenotype during terminal differentiation [59]. This effect is mediated by Smad signaling and might be in part due to suppression of lipolysis, via regulation of hormone-sensitive lipase expression linked to reduced PPAR activity [59]. Also, in humans, there is a strong correlation between BMP4 levels and adipocyte size [59].

However, findings contrast with the above research that suggested that BMP4 has effects on white-to-brown transition in primary human adipose stem cells (hASCs) from subcutaneous AT [60]. BMP4 increased UCP1 expression in hASCs and decreased expression of the white-specific marker TCF21 [60]. Adding BMP4 during white adipogenic differentiation reactivated beige/brown markers [58]. BMP4 could also induce brown fat-like adipocytes in both white and brown preadipocytes, thereby decreasing the classical brown adipocyte marker Zic1 and increasing the recently identified beige adipocyte marker TMEM26 [61]. BMP4 promotes brown adipocyte differentiation and thermogenesis in vivo and in vitro which offers a potentially new therapeutic approach for the treatment of obesity [61]. More studies are warranted to prove it.

### 4.3. BMP2

BMP2 may contribute to the partition of energy storage into visceral (VAT) and subcutaneous adipose tissue (SAT) [10]. BMP2 expression in both VAT and SAT was significantly higher in people with obesity when compared with individuals who were healthy and lean [10]. BMP2 mRNA was significantly higher in VAT compared with SAT in 547 individuals with a wide range of body mass index (BMI) [10]. Recent large-scale genome-wide association studies (GWAS) have identified a genetic variant rs979012 within BMP2 which was associated with the waist-to-hip ratio (WHR). That may be a mechanism of BMP2 in the regulation of fat distribution.

Besides, BMP2 is a potential plasma indicator of inflammatory status in middle-aged and elderly women [62]. The increased cardiovascular risk was positively correlated with BMP2 levels in overweight and obese middle-aged and elderly individuals [62].

### 4.4. BMP7

#### 4.4.1. BMP7 and Adipose Tissue

**Figure 3:**
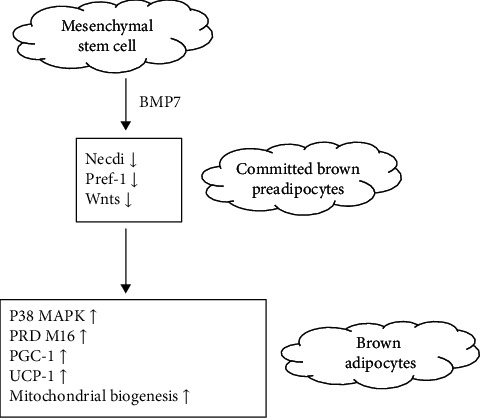
BMP7 can regulate brown adipogenesis and energy expenditure.

BMP7 may be a promising tool for the treatment for obesity and associated comorbidities. Adipose tissue is central to the regulation of energy balance. WAT is the primary site of triglyceride storage while BAT and beige adipocytes are enriched in mitochondria with UCP1 to generate heat instead of ATP contributing to healthy energy balance. BMP7 is identified as an inducer of BAT differentiation [63, 64]. BMP7 can regulate brown adipogenesis and energy expenditure through a leptin-independent pathway [65] [64, 66]. BMP7 can regulate brown adipogenesis and energy expenditure through some factors, such as P38 MAPK, PRD M16, PGC-1, UCP-1, and mitochondrial biogenesis. Also, it affects browning [67] as presented in Figure 3. The autocrine mediator BMP7 led to moderate browning with the upregulation of the classical brown marker Zic1 instead of Tbx1 targets for enhancing thermogenesis in obesity [68]. Adenoviral-mediated expression of BMP7 in mice leads to a significant increase in BAT, but not WAT, and results in an increase in energy expenditure and a reduction in weight gain [65].

#### 4.4.2. BMP7 and Appetite

Energy intake and energy expenditure coordinate body weight. BMP7 exerts new function in appetite regulation [69]. Systemic treatment of diet-induced obese mice and ob/ob mice with BMP7 leads to a significantly decreased body weight by increasing energy expenditure and decreasing food intake and improved metabolism by a leptin-independent mechanism, at least partly by a central rapamycin-sensitive mTOR-p70S6 kinase pathway [69].

In a word, changes in a range of related factors are caused by metabolic disorders in the obesity including the BMP family. Studying the BMP family provides a better understanding of the pathophysiological mechanisms of obesity.

## 5. BMPs and Glucose Metabolism

### 5.1. BMP9

Plasma BMP9 is independently negatively correlated with insulin resistance (IR) assessed by homeostasis model assessment of insulin resistance (HOMA-IR) in Mets and newly diagnosed patients with T2DM [31] [9]. Furthermore, BMP9 mRNA and protein expressions were significantly decreased in muscle and adipose tissues of T2DM patients which suggests that BMP9 is likely to play an important role in insulin resistance [31].

In vitro, hepatic BMP9 expression was downregulated in IR mice and the overexpression of hepatic BMP9 improved IR in mice with HFD [30]. The level of insulin signaling molecule phosphorylation was increased in the livers of adenovirus-BMP9-treated mice and glucosamine-treated hepatocytes [30].

BMP9 whose expression is highest in the liver cell is likely to play an important role in glucose metabolism which is independently associated with T2DM [9, 31, 70]. In humans, hepatic BMP9 expression was downregulated in patients with diabetes [30]. Additionally, circulating BMP9 levels were significantly higher in healthy subjects than in newly diagnosed patients with T2DM [31]. Besides, it negatively correlated with markers of glucose metabolism including HbA1c, fasting blood glucose (FBG), OGTT, and the area under the curve for glucose (AUC_glucose_) [31]. Also, plasma BMP9 is associated negatively with FBG, 2-hour blood glucose after glucose overload (2 h-OGTT), and HbA1c in 362 newly diagnosed patients with Mets [9]. BMP9 is an important regulator of hepatic glucose metabolism [30]. The overexpression of hepatic BMP9 improved glucose tolerance in mice fed with HFD [30]. The expression of gluconeogenic genes was downregulated in the livers of adenovirus-BMP9-treated mice and glucosamine-treated hepatocytes [30]. BMP9 enhances fatty acid synthase expression in the liver of obese mice, which may improve insulin resistance and help to attenuate blood glucose levels [53].

BMP9 can regulate glucose by inhibiting liver gluconeogenesis, transforming white adipose tissue to brown adipose tissue, promoting muscle glycogen synthesis, increasing the uptake and utilization of glucose by muscle tissue, increasing liver and adipose tissue insulin sensitivity, and promoting insulin synthesis and secretion [71]. It has been confirmed that BMP9, like insulin, improves glycemia in diabetic mice and regulates directional glucose metabolism in hepatocytes. Therefore, the hypoglycemic potential of BMP9 is of great interest. It is proposed to be a candidate hepatic insulin-sensitizing substance (HISS) [72, 73].

### 5.2. BMP4

Moreover, increasing studies have demonstrated the effects of BMP4 in both bone and glucose metabolism [58, 59, 74, 75]. Hyperglycemia and free fatty acid stimulate BMP4 expression [76]. BMP4 is upregulated in diabetic animals with inhibition of glucose-stimulated insulin secretion in rodent pancreatic islets in a calbindin 1-dependent manner [77]. A study from Christensen et al. shows that prolonged exposure to BMP4 reduced glucose-stimulated insulin secretion in rodent pancreatic islets. BMP4 can significantly induce the expression of the Ca (2+)-binding protein calbindin 1 and reduce Ca (2+) current through voltage-dependent Ca (2+) channels. The decrease in Ca (2+) channel activity leads to diminished insulin exocytosis.

BMP4 plays a role in the regulation of glucose homeostasis by inhibiting insulin signaling via activation of PKC-*θ* isoform, which results in insulin resistance [78]. BMP4 levels are significantly associated with insulin sensitivity in humans [59].

### 5.3. BMP2

BMP2 serum concentrations were significantly higher in patients with T2DM [10]. The BMP2 pathway may be a promising new drug target to treat IR with insulin-sensitizing effect by enhancing insulin-mediated glucose uptake in both insulin-sensitive and insulin-insensitive adipocytes [11]. BMP2 has a direct effect on the translocation of the GLUT4 to the plasma membrane and demonstrates that these BMP2 increase GLUT4 protein levels equipotent to rosiglitazone [11]. Also, BMP2 is a potential therapeutic target to prevent or treat diabetic retinopathy. A recent in vitro study found that BMP2 impacts Vascular Endothelial Growth Factor (VEGF) expression in cultured Müller cells. VEGF has always been regarded as a key player in the pathogenesis and progression of DR due to its potent proangiogenic and proinflammatory effects. This finding suggests that the effect was not linked to glycemic balance [79].

### 5.4. BMP7

BMP7 provides new insights into treating insulin resistance-related disorders such as T2DM. BMP7 regulates hepatic insulin sensitivity. Ma et al. found that hepatic BMP7 expression is reduced in HFD-induced diabetic mice and palmitate-induced insulin-resistant HepG2 and AML12 cells [80]. BMP7 improves the insulin signaling pathway in IR hepatocytes while knockdown of BMP7 further impairs insulin signal transduction in PA-treated cells [80]. Both in vitro and in vivo, the study showed that hepatic BMP7 has a novel function in regulating insulin sensitivity through inhibition of mitogen-activated protein kinases (MAPKs) in both the liver of obese mice and PA-treated cells [80]. BMP7 is an attractive candidate for tackling diabetes. It causes improved glucose uptake and ameliorates peripheral IR by potentiating insulin signaling of the PI3K/AKT pathway in mice with T2DM [78]. BMP7 may stimulate insulin secretion and improve islet cell function in nondiabetic individuals, with the results showing that serum BMP7 concentrations were positively correlated with HOMA-B (insulin secretion index) and fasting insulin [81].

### 5.5. BMP6

BMP6 is efficient in bone formation [82–84]. BMP6 improves glycemia in T2D mice and regulates glucose metabolism in hepatocytes. Treatment of ob/ob mice with BMP6 for 6 days resulted in reduced circulating glucose and lipid levels [85]. BMP6 improved the glucose excursion during an OGTT, lowering the total glycemic response by 21% [85]. BMP6 inhibited gluconeogenesis and glucose output via downregulating the PepCK expression in rat H4IIE hepatoma cells [85]. Moreover, BMP6 inhibited glucose production regardless of the presence of cAMP [85]. Besides, the BMP6 pathway is a promising new drug target to treat IR [11]. BMP6 leads to enhanced insulin-mediated glucose uptake in both insulin-sensitive and insulin-insensitive adipocytes [11]. BMP6 has a direct effect on the translocation of GLUT4 to the plasma membrane and demonstrates that these BMP6 increase GLUT4 protein levels equal to rosiglitazone [11]. In addition, BMP6 may provide a means to enhance the amount of myogenic lineage-derived BAT, which plays a pivotal role in promoting energy expenditure [84, 86]. BMP6 might serve as a novel therapeutic for obesity by enhancing the amount of myogenic lineage-derived BAT [84, 86]. Therefore, the BMP family may be an exciting prospect for future treatments of diabetes as BMP2, BMP6, BMP7, and BMP 9 have function of improving glucose metabolism.

## 6. Conclusion

Overall, BMPs, mainly including BMP9, BMP4, BMP2, and BMP7, may exert functions both in bone and in obesity as well as glucose metabolism. A full understanding of the effects of BMPs may help to better understand the bone metabolism in obesity as well as type 2 diabetes. The BMPs may play a role as a bridge among bone, obesity, and glucose metabolism.

## Abbreviations

T2DM: Type 2 diabetes
BMD: Bone mineral density
BMPs: Bone morphogenetic proteins
TGF: Transforming growth factor
MMCs: Murine multilineage cells
GDF-2: Differentiation factor
NF-*κ*B: Nuclear factor-*κ*B
Mets: Metabolic syndrome
WHR: Waist-hip ratio
FGF21: Fibroblast growth factor 21
HFD: High-fat diet
NAFLD: Nonalcoholic fatty liver disease
ALT: Aminotransferase
BAT: Brown adipose tissue
UCP1: Uncoupling protein 1
WAT: White adipose tissue.
